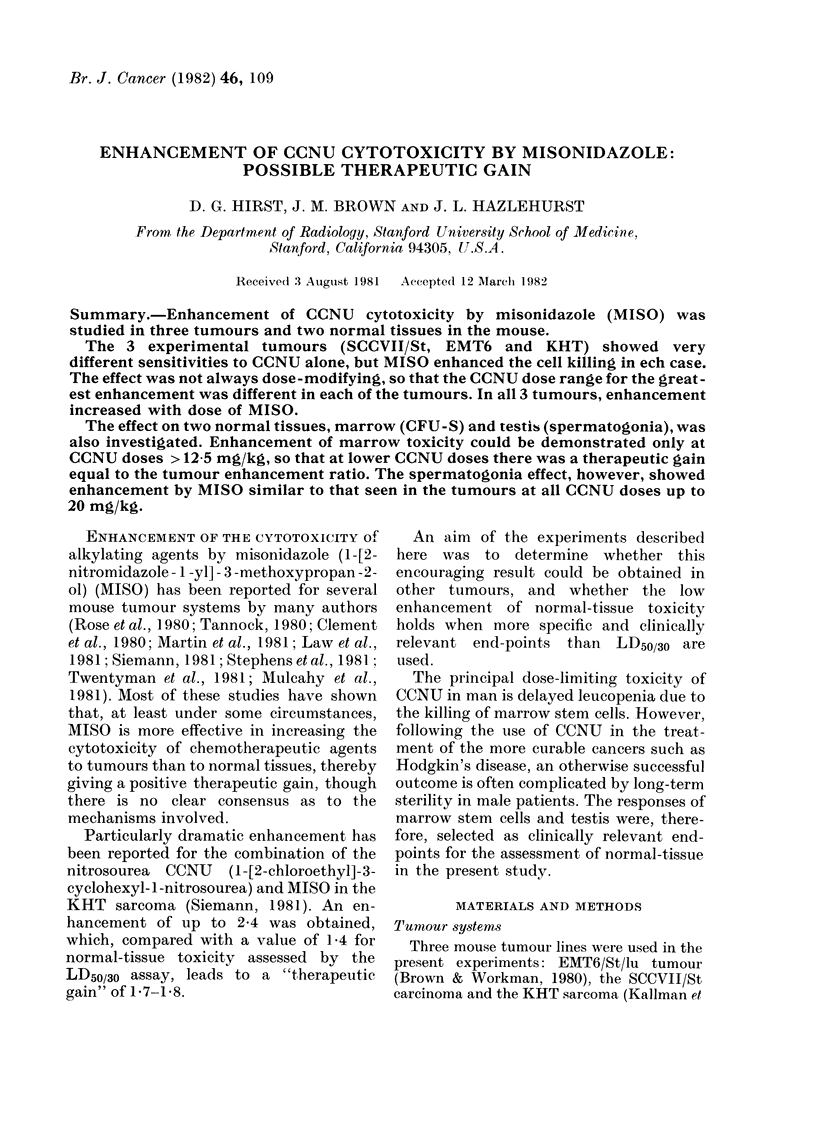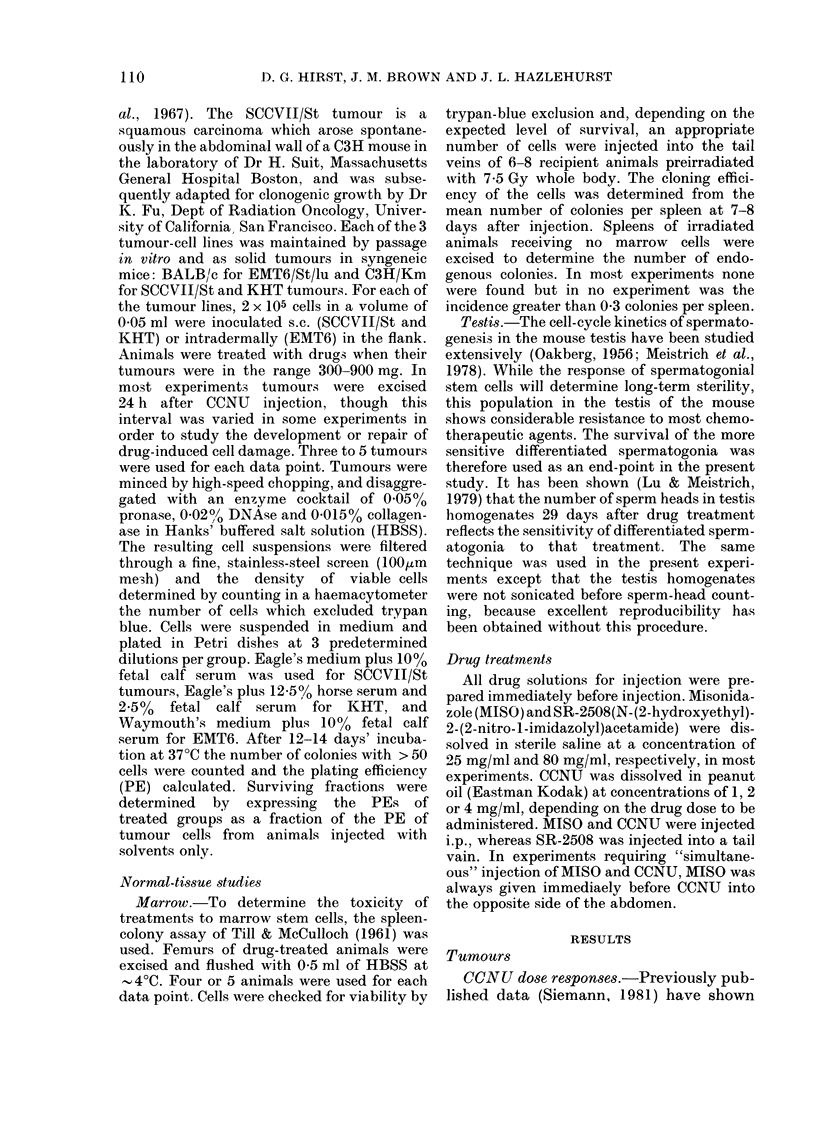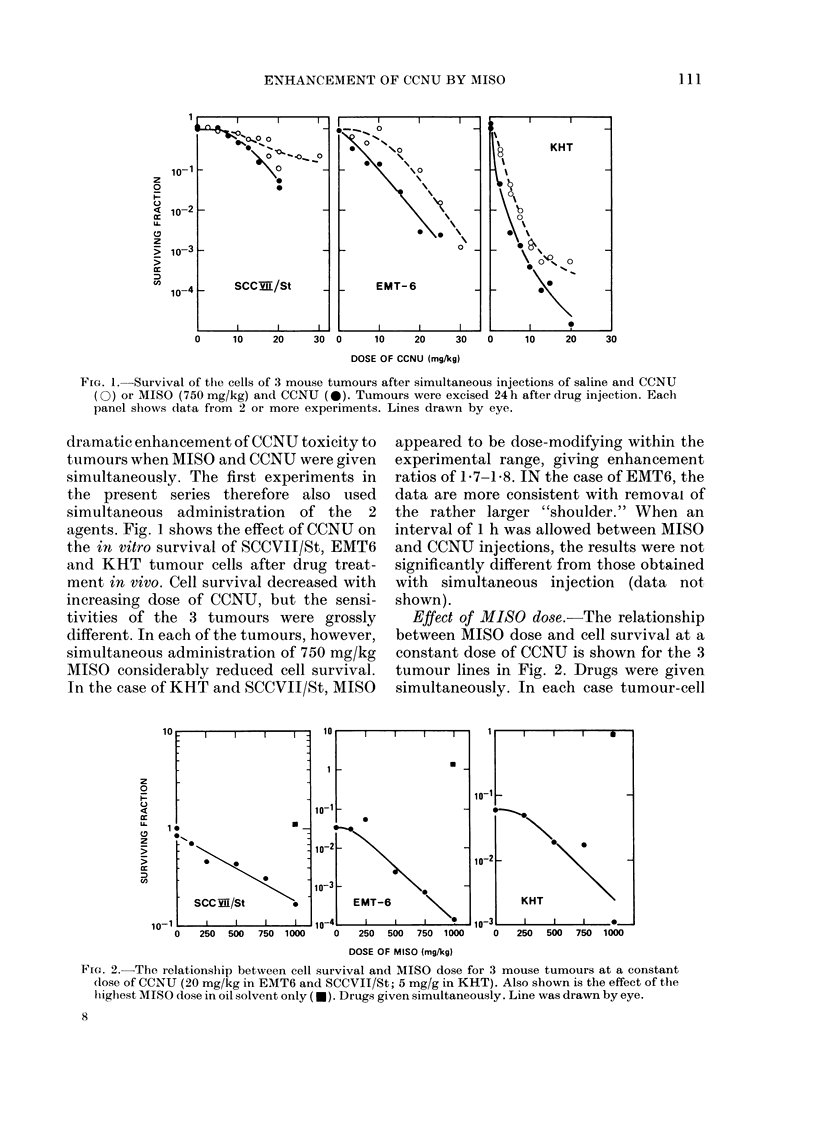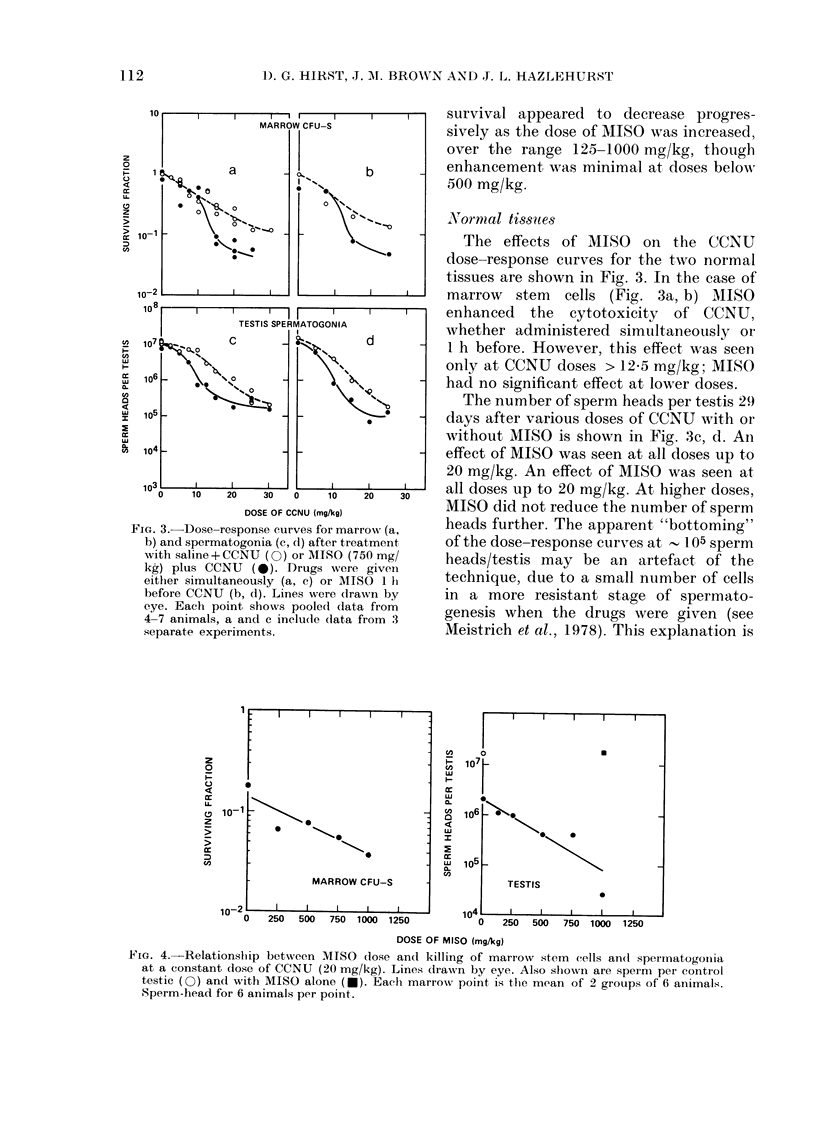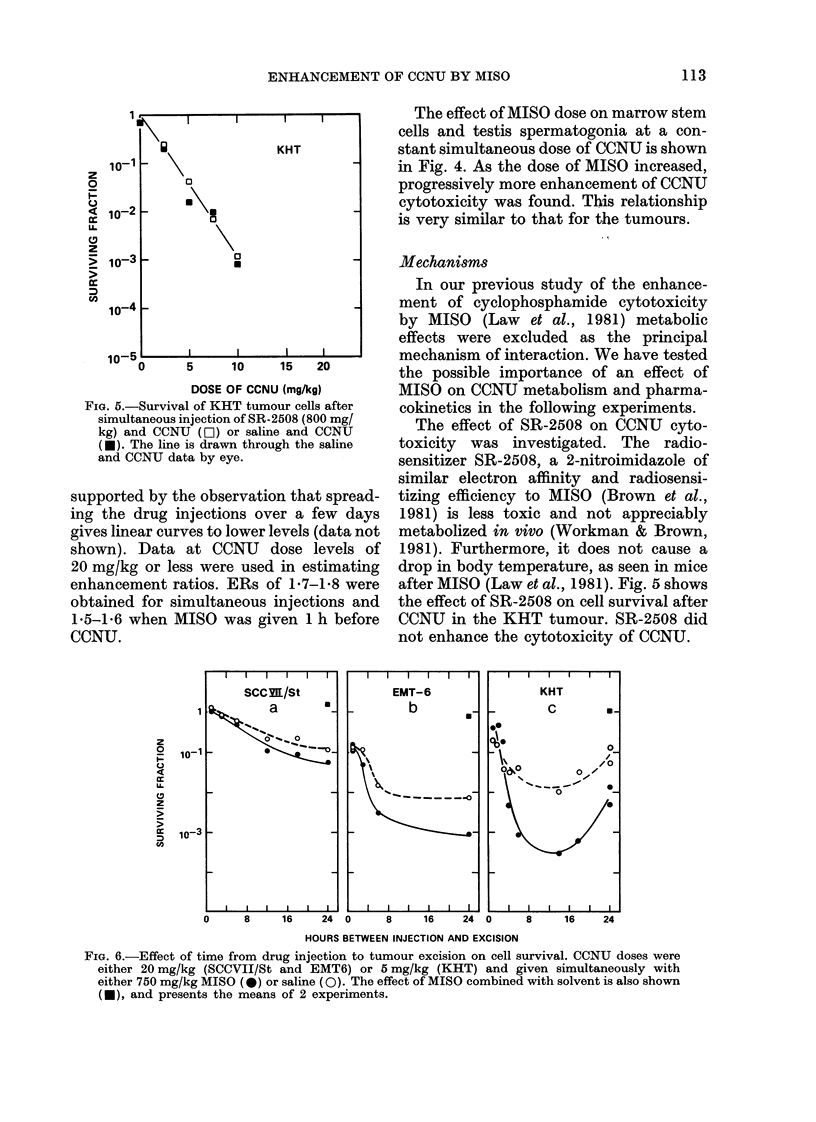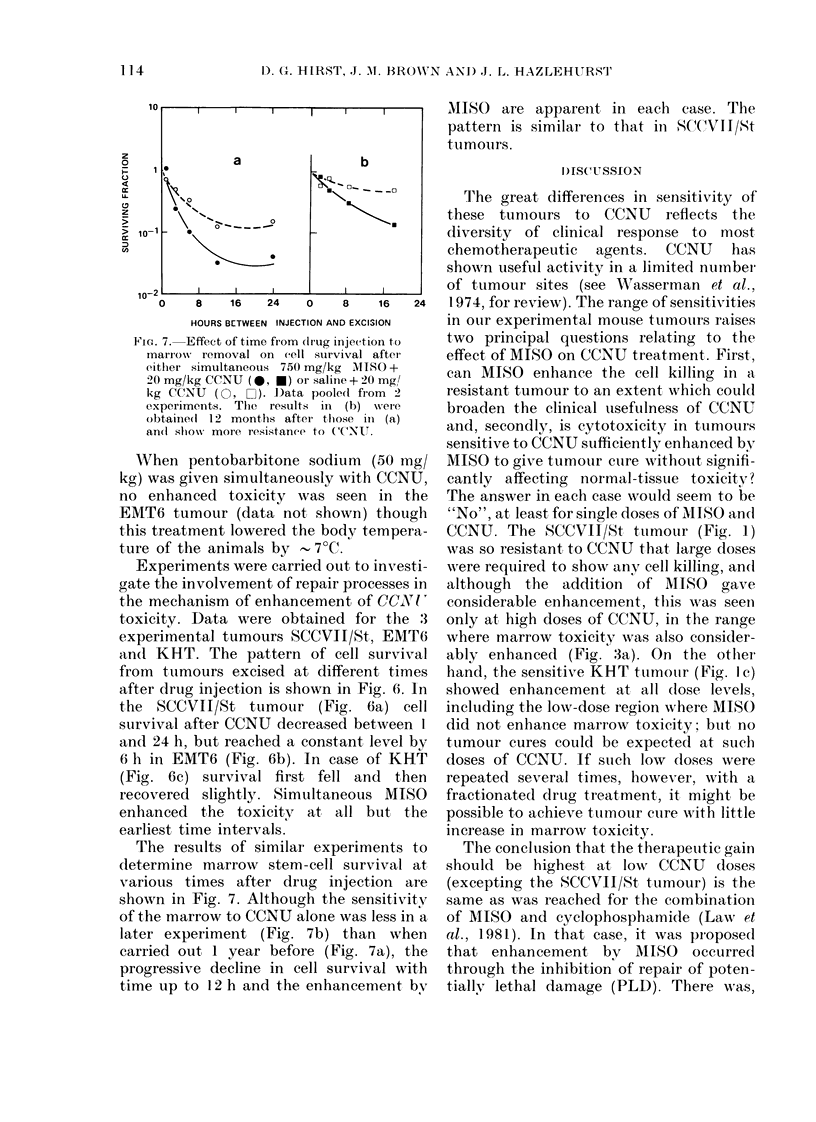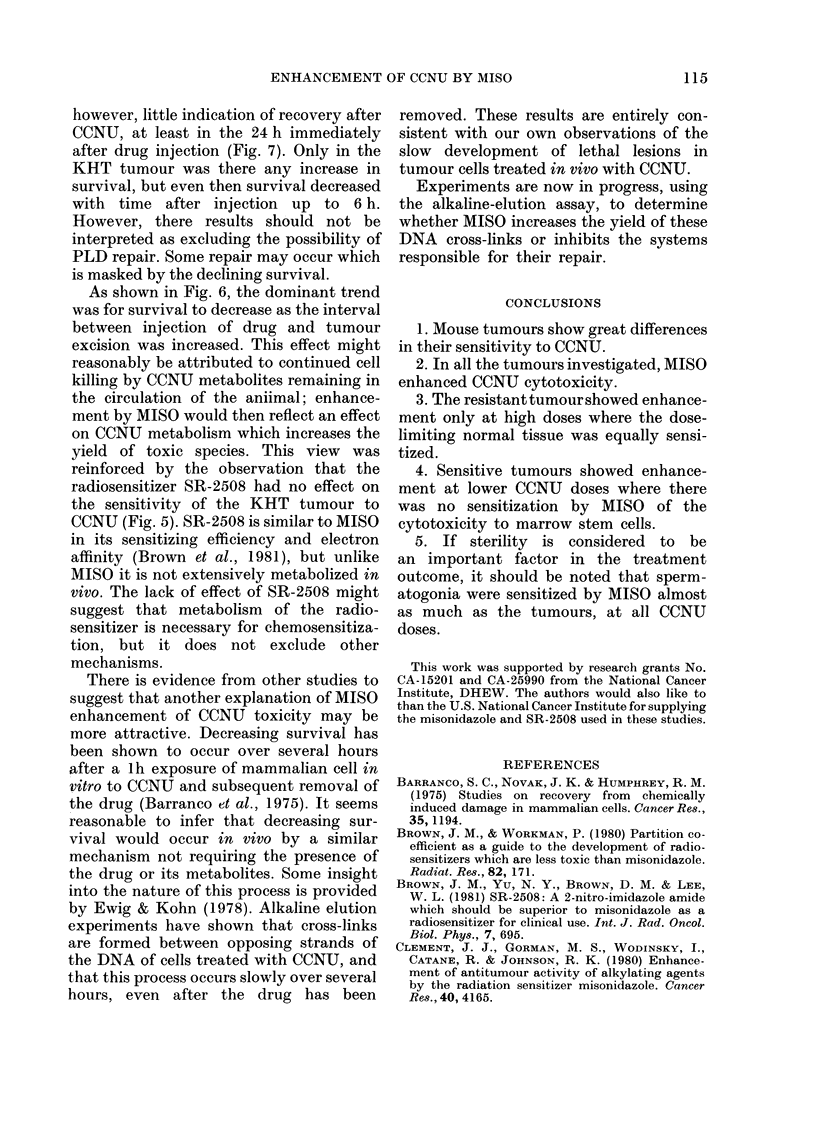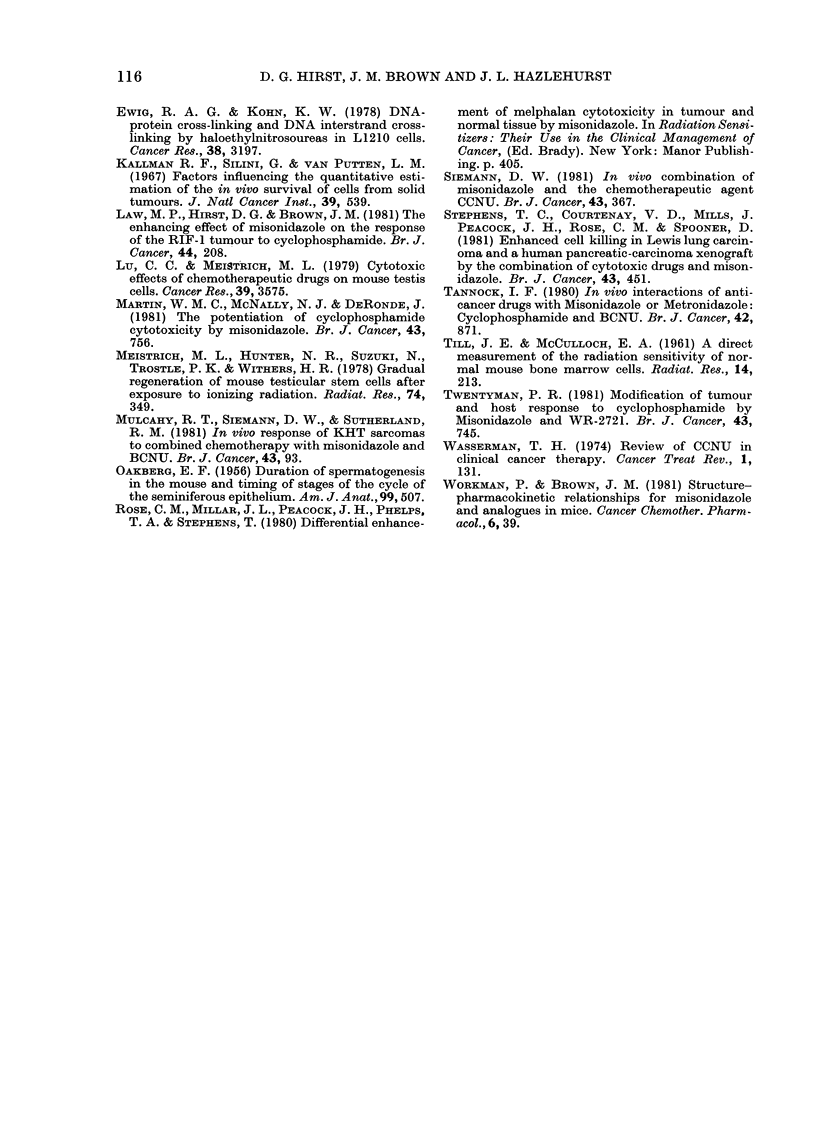# Enhancement of CCNU cytotoxicity by misonidazole: possible therapeutic gain.

**DOI:** 10.1038/bjc.1982.172

**Published:** 1982-07

**Authors:** D. G. Hirst, J. M. Brown, J. L. Hazlehurst

## Abstract

Enhancement of CCNU cytotoxicity by misonidazole (MISO) was studied in three tumours and two normal tissues in the mouse. The 3 experimental tumours (SCCVII/St, EMT6 and KHT) showed very different sensitivities to CCNU alone, but MISO enhanced the cell killing in ech case. The effect was not always dose-modifying, so that the CCNU dose range for the greatest enhancement was different in each of the tumours. In all 3 tumours, enhancement increased with dose of MISO. The effect on two normal tissues, marrow (CFU-S) and testis (spermatogonia), was also investigated. Enhancement of marrow toxicity could be demonstrated only at CCNU doses greater than 12.5 mg/kg, so that at lower CCNU doses there was a therapeutic gain equal to the tumour enhancement ratio. The spermatogonia effect, however, showed enhancement by MISO similar to that seen in the tumours at all CCNU doses up to 20 mg/kg.


*Br. J. Cancer* (1982) **46**, 109

### ENHANCEMENT OF CCNU CYTOTOXICITY BY MISONIDAZOLE: POSSIBLE THERAPEUTIC GAIN

D. G. HIRST, J. M. BROWN AND J. L. HAZLEHURST

*From the Department of Radiology, Stanford University School of Medicine, Stanford, California* 94305, *U.S.A.*

Received 3 August 1981 Accepted 12 March 1982

**Summary.—Enhancement of CCNU cytotoxicity by misonidazole (MISO) was studied in three tumours and two normal tissues in the mouse.**

**The 3 experimental tumours (SCCVII/St, EMT6 and KHT) showed very different sensitivities to CCNU alone, but MISO enhanced the cell killing in ech case. The effect was not always dose-modifying, so that the CCNU dose range for the greatest enhancement was different in each of the tumours. In all 3 tumours, enhancement increased with dose of MISO.**

**The effect on two normal tissues, marrow (CFU-S) and testis (spermatogonia), was also investigated. Enhancement of marrow toxicity could be demonstrated only at CCNU doses >12·5 mg/kg, so that at lower CCNU doses there was a therapeutic gain equal to the tumour enhancement ratio. The spermatogonia effect, however, showed enhancement by MISO similar to that seen in the tumours at all CCNU doses up to 20 mg/kg.**

ENHANCEMENT OF THE CYTOTOXICITY of alkylating agents by misonidazole (1-[2-nitromidazole-1-yl]-3-methoxypropan-2-ol) (MISO) has been reported for several mouse tumour systems by many authors (Rose *et al.*, 1980; Tannock, 1980; Clement *et al.*, 1980; Martin *et al.*, 1981; Law *et al.*, 1981; Siemann, 1981; Stephens *et al.*, 1981; Twentyman *et al.*, 1981; Mulcahy *et al.*, 1981). Most of these studies have shown that, at least under some circumstances, MISO is more effective in increasing the cytotoxicity of chemotherapeutic agents to tumours than to normal tissues, thereby giving a positive therapeutic gain, though there is no clear consensus as to the mechanisms involved.

Particularly dramatic enhancement has been reported for the combination of the nitrosourea CCNU (1-[2-chloroethyl]-3-cyclohexyl-1-nitrosourea) and MISO in the KHT sarcoma (Siemann, 1981). An enhancement of up to 2·4 was obtained, which, compared with a value of 1·4 for normal-tissue toxicity assessed by the $LD_{50/30}$ assay, leads to a "therapeutic gain" of 1·7–1·8.

An aim of the experiments described here was to determine whether this encouraging result could be obtained in other tumours, and whether the low enhancement of normal-tissue toxicity holds when more specific and clinically relevant end-points than $LD_{50/30}$ are used.

The principal dose-limiting toxicity of CCNU in man is delayed leucopenia due to the killing of marrow stem cells. However, following the use of CCNU in the treatment of the more curable cancers such as Hodgkin's disease, an otherwise successful outcome is often complicated by long-term sterility in male patients. The responses of marrow stem cells and testis were, therefore, selected as clinically relevant end-points for the assessment of normal-tissue in the present study.

### MATERIALS AND METHODS

### *Tumour systems*

Three mouse tumour lines were used in the present experiments: EMT6/St/lu tumour (Brown & Workman, 1980), the SCCVII/St carcinoma and the KHT sarcoma (Kallman *et*

*al.*, 1967). The SCCVII/St tumour is a squamous carcinoma which arose spontaneously in the abdominal wall of a C3H mouse in the laboratory of Dr H. Suit, Massachusetts General Hospital Boston, and was subsequently adapted for clonogenic growth by Dr K. Fu, Dept of Radiation Oncology, University of California, San Francisco. Each of the 3 tumour-cell lines was maintained by passage *in vitro* and as solid tumours in syngeneic mice: BALB/c for EMT6/St/lu and C3H/Km for SCCVII/St and KHT tumours. For each of the tumour lines, $2 \times 10^5$ cells in a volume of 0·05 ml were inoculated s.c. (SCCVII/St and KHT) or intradermally (EMT6) in the flank. Animals were treated with drugs when their tumours were in the range 300–900 mg. In most experiments tumours were excised 24 h after CCNU injection, though this interval was varied in some experiments in order to study the development or repair of drug-induced cell damage. Three to 5 tumours were used for each data point. Tumours were minced by high-speed chopping, and disaggregated with an enzyme cocktail of 0·05% pronase, 0·02% DNAse and 0·015% collagenase in Hanks' buffered salt solution (HBSS). The resulting cell suspensions were filtered through a fine, stainless-steel screen (100 $\mu$m mesh) and the density of viable cells determined by counting in a haemacytometer the number of cells which excluded trypan blue. Cells were suspended in medium and plated in Petri dishes at 3 predetermined dilutions per group. Eagle's medium plus 10% fetal calf serum was used for SCCVII/St tumours, Eagle's plus 12·5% horse serum and 2·5% fetal calf serum for KHT, and Waymouth's medium plus 10% fetal calf serum for EMT6. After 12–14 days' incubation at 37°C the number of colonies with $>50$ cells were counted and the plating efficiency (PE) calculated. Surviving fractions were determined by expressing the PEs of treated groups as a fraction of the PE of tumour cells from animals injected with solvents only.

*Normal-tissue studies*

*Marrow.*—To determine the toxicity of treatments to marrow stem cells, the spleen-colony assay of Till & McCulloch (1961) was used. Femurs of drug-treated animals were excised and flushed with 0·5 ml of HBSS at $\sim$4°C. Four or 5 animals were used for each data point. Cells were checked for viability by trypan-blue exclusion and, depending on the expected level of survival, an appropriate number of cells were injected into the tail veins of 6–8 recipient animals preirradiated with 7·5 Gy whole body. The cloning efficiency of the cells was determined from the mean number of colonies per spleen at 7–8 days after injection. Spleens of irradiated animals receiving no marrow cells were excised to determine the number of endogenous colonies. In most experiments none were found but in no experiment was the incidence greater than 0·3 colonies per spleen.

*Testis.*—The cell-cycle kinetics of spermatogenesis in the mouse testis have been studied extensively (Oakberg, 1956; Meistrich *et al.*, 1978). While the response of spermatogonial stem cells will determine long-term sterility, this population in the testis of the mouse shows considerable resistance to most chemotherapeutic agents. The survival of the more sensitive differentiated spermatogonia was therefore used as an end-point in the present study. It has been shown (Lu & Meistrich, 1979) that the number of sperm heads in testis homogenates 29 days after drug treatment reflects the sensitivity of differentiated spermatogonia to that treatment. The same technique was used in the present experiments except that the testis homogenates were not sonicated before sperm-head counting, because excellent reproducibility has been obtained without this procedure.

*Drug treatments*

All drug solutions for injection were prepared immediately before injection. Misonidazole (MISO) and SR-2508 (N-(2-hydroxyethyl)-2-(2-nitro-1-imidazolyl)acetamide) were dissolved in sterile saline at a concentration of 25 mg/ml and 80 mg/ml, respectively, in most experiments. CCNU was dissolved in peanut oil (Eastman Kodak) at concentrations of 1, 2 or 4 mg/ml, depending on the drug dose to be administered. MISO and CCNU were injected i.p., whereas SR-2508 was injected into a tail vain. In experiments requiring "simultaneous" injection of MISO and CCNU, MISO was always given immediaely before CCNU into the opposite side of the abdomen.

### RESULTS

*Tumours*

*CCNU dose responses.*—Previously published data (Siemann, 1981) have shown

FIG. 1.—Survival of the cells of 3 mouse tumours after simultaneous injections of saline and CCNU (○) or MISO (750 mg/kg) and CCNU (●). Tumours were excised 24 h after drug injection. Each panel shows data from 2 or more experiments. Lines drawn by eye.

dramatic enhancement of CCNU toxicity to tumours when MISO and CCNU were given simultaneously. The first experiments in the present series therefore also used simultaneous administration of the 2 agents. Fig. 1 shows the effect of CCNU on the *in vitro* survival of SCCVII/St, EMT6 and KHT tumour cells after drug treatment *in vivo*. Cell survival decreased with increasing dose of CCNU, but the sensitivities of the 3 tumours were grossly different. In each of the tumours, however, simultaneous administration of 750 mg/kg MISO considerably reduced cell survival. In the case of KHT and SCCVII/St, MISO appeared to be dose-modifying within the experimental range, giving enhancement ratios of 1·7–1·8. IN the case of EMT6, the data are more consistent with removal of the rather larger "shoulder." When an interval of 1 h was allowed between MISO and CCNU injections, the results were not significantly different from those obtained with simultaneous injection (data not shown).

*Effect of MISO dose.*—The relationship between MISO dose and cell survival at a constant dose of CCNU is shown for the 3 tumour lines in Fig. 2. Drugs were given simultaneously. In each case tumour-cell

FIG. 2.—The relationship between cell survival and MISO dose for 3 mouse tumours at a constant dose of CCNU (20 mg/kg in EMT6 and SCCVII/St; 5 mg/g in KHT). Also shown is the effect of the highest MISO dose in oil solvent only (■). Drugs given simultaneously. Line was drawn by eye.

Fig. 3.—Dose–response curves for marrow (a, b) and spermatogonia (c, d) after treatment with saline + CCNU (○) or MISO (750 mg/kg) plus CCNU (●). Drugs were given either simultaneously (a, c) or MISO 1 h before CCNU (b, d). Lines were drawn by eye. Each point shows pooled data from 4–7 animals, a and c include data from 3 separate experiments.

survival appeared to decrease progressively as the dose of MISO was increased, over the range 125–1000 mg/kg, though enhancement was minimal at doses below 500 mg/kg.

*Normal tissues*

The effects of MISO on the CCNU dose–response curves for the two normal tissues are shown in Fig. 3. In the case of marrow stem cells (Fig. 3a, b) MISO enhanced the cytotoxicity of CCNU, whether administered simultaneously or 1 h before. However, this effect was seen only at CCNU doses $> 12·5$ mg/kg; MISO had no significant effect at lower doses.

The number of sperm heads per testis 29 days after various doses of CCNU with or without MISO is shown in Fig. 3c, d. An effect of MISO was seen at all doses up to 20 mg/kg. An effect of MISO was seen at all doses up to 20 mg/kg. At higher doses, MISO did not reduce the number of sperm heads further. The apparent "bottoming" of the dose–response curves at $\sim 10^5$ sperm heads/testis may be an artefact of the technique, due to a small number of cells in a more resistant stage of spermatogenesis when the drugs were given (see Meistrich *et al.*, 1978). This explanation is

Fig. 4.—Relationship between MISO dose and killing of marrow stem cells and spermatogonia at a constant dose of CCNU (20 mg/kg). Lines drawn by eye. Also shown are sperm per control testic (○) and with MISO alone (■). Each marrow point is the mean of 2 groups of 6 animals. Sperm-head for 6 animals per point.

FIG. 5.—Survival of KHT tumour cells after simultaneous injection of SR-2508 (800 mg/kg) and CCNU (□) or saline and CCNU (■). The line is drawn through the saline and CCNU data by eye.

supported by the observation that spreading the drug injections over a few days gives linear curves to lower levels (data not shown). Data at CCNU dose levels of 20 mg/kg or less were used in estimating enhancement ratios. ERs of 1·7–1·8 were obtained for simultaneous injections and 1·5–1·6 when MISO was given 1 h before CCNU.

The effect of MISO dose on marrow stem cells and testis spermatogonia at a constant simultaneous dose of CCNU is shown in Fig. 4. As the dose of MISO increased, progressively more enhancement of CCNU cytotoxicity was found. This relationship is very similar to that for the tumours.

*Mechanisms*

In our previous study of the enhancement of cyclophosphamide cytotoxicity by MISO (Law *et al.*, 1981) metabolic effects were excluded as the principal mechanism of interaction. We have tested the possible importance of an effect of MISO on CCNU metabolism and pharmacokinetics in the following experiments.

The effect of SR-2508 on CCNU cytotoxicity was investigated. The radiosensitizer SR-2508, a 2-nitroimidazole of similar electron affinity and radiosensitizing efficiency to MISO (Brown *et al.*, 1981) is less toxic and not appreciably metabolized *in vivo* (Workman & Brown, 1981). Furthermore, it does not cause a drop in body temperature, as seen in mice after MISO (Law *et al.*, 1981). Fig. 5 shows the effect of SR-2508 on cell survival after CCNU in the KHT tumour. SR-2508 did not enhance the cytotoxicity of CCNU.

FIG. 6.—Effect of time from drug injection to tumour excision on cell survival. CCNU doses were either 20 mg/kg (SCCVII/St and EMT6) or 5 mg/kg (KHT) and given simultaneously with either 750 mg/kg MISO (●) or saline (○). The effect of MISO combined with solvent is also shown (■), and presents the means of 2 experiments.

FIG. 7.—Effect of time from drug injection to marrow removal on cell survival after either simultaneous 750 mg/kg MISO + 20 mg/kg CCNU (●, ■) or saline + 20 mg/kg CCNU (○, □). Data pooled from 2 experiments. The results in (b) were obtained 12 months after those in (a) and show more resistance to CCNU.

When pentobarbitone sodium (50 mg/kg) was given simultaneously with CCNU, no enhanced toxicity was seen in the EMT6 tumour (data not shown) though this treatment lowered the body temperature of the animals by ~7°C.

Experiments were carried out to investigate the involvement of repair processes in the mechanism of enhancement of *CCNU* toxicity. Data were obtained for the 3 experimental tumours SCCVII/St, EMT6 and KHT. The pattern of cell survival from tumours excised at different times after drug injection is shown in Fig. 6. In the SCCVII/St tumour (Fig. 6a) cell survival after CCNU decreased between 1 and 24 h, but reached a constant level by 6 h in EMT6 (Fig. 6b). In case of KHT (Fig. 6c) survival first fell and then recovered slightly. Simultaneous MISO enhanced the toxicity at all but the earliest time intervals.

The results of similar experiments to determine marrow stem-cell survival at various times after drug injection are shown in Fig. 7. Although the sensitivity of the marrow to CCNU alone was less in a later experiment (Fig. 7b) than when carried out 1 year before (Fig. 7a), the progressive decline in cell survival with time up to 12 h and the enhancement by MISO are apparent in each case. The pattern is similar to that in SCCVII/St tumours.

### DISCUSSION

The great differences in sensitivity of these tumours to CCNU reflects the diversity of clinical response to most chemotherapeutic agents. CCNU has shown useful activity in a limited number of tumour sites (see Wasserman *et al.*, 1974, for review). The range of sensitivities in our experimental mouse tumours raises two principal questions relating to the effect of MISO on CCNU treatment. First, can MISO enhance the cell killing in a resistant tumour to an extent which could broaden the clinical usefulness of CCNU and, secondly, is cytotoxicity in tumours sensitive to CCNU sufficiently enhanced by MISO to give tumour cure without significantly affecting normal-tissue toxicity? The answer in each case would seem to be "No", at least for single doses of MISO and CCNU. The SCCVII/St tumour (Fig. 1) was so resistant to CCNU that large doses were required to show any cell killing, and although the addition of MISO gave considerable enhancement, this was seen only at high doses of CCNU, in the range where marrow toxicity was also considerably enhanced (Fig. 3a). On the other hand, the sensitive KHT tumour (Fig. 1c) showed enhancement at all dose levels, including the low-dose region where MISO did not enhance marrow toxicity; but no tumour cures could be expected at such doses of CCNU. If such low doses were repeated several times, however, with a fractionated drug treatment, it might be possible to achieve tumour cure with little increase in marrow toxicity.

The conclusion that the therapeutic gain should be highest at low CCNU doses (excepting the SCCVII/St tumour) is the same as was reached for the combination of MISO and cyclophosphamide (Law *et al.*, 1981). In that case, it was proposed that enhancement by MISO occurred through the inhibition of repair of potentially lethal damage (PLD). There was, however, little indication of recovery after CCNU, at least in the 24 h immediately after drug injection (Fig. 7). Only in the KHT tumour was there any increase in survival, but even then survival decreased with time after injection up to 6 h. However, there results should not be interpreted as excluding the possibility of PLD repair. Some repair may occur which is masked by the declining survival.

As shown in Fig. 6, the dominant trend was for survival to decrease as the interval between injection of drug and tumour excision was increased. This effect might reasonably be attributed to continued cell killing by CCNU metabolites remaining in the circulation of the aniimal; enhancement by MISO would then reflect an effect on CCNU metabolism which increases the yield of toxic species. This view was reinforced by the observation that the radiosensitizer SR-2508 had no effect on the sensitivity of the KHT tumour to CCNU (Fig. 5). SR-2508 is similar to MISO in its sensitizing efficiency and electron affinity (Brown *et al.*, 1981), but unlike MISO it is not extensively metabolized *in vivo*. The lack of effect of SR-2508 might suggest that metabolism of the radiosensitizer is necessary for chemosensitization, but it does not exclude other mechanisms.

There is evidence from other studies to suggest that another explanation of MISO enhancement of CCNU toxicity may be more attractive. Decreasing survival has been shown to occur over several hours after a 1h exposure of mammalian cell *in vitro* to CCNU and subsequent removal of the drug (Barranco *et al.*, 1975). It seems reasonable to infer that decreasing survival would occur *in vivo* by a similar mechanism not requiring the presence of the drug or its metabolites. Some insight into the nature of this process is provided by Ewig & Kohn (1978). Alkaline elution experiments have shown that cross-links are formed between opposing strands of the DNA of cells treated with CCNU, and that this process occurs slowly over several hours, even after the drug has been removed. These results are entirely consistent with our own observations of the slow development of lethal lesions in tumour cells treated *in vivo* with CCNU.

Experiments are now in progress, using the alkaline-elution assay, to determine whether MISO increases the yield of these DNA cross-links or inhibits the systems responsible for their repair.

### CONCLUSIONS

1. Mouse tumours show great differences in their sensitivity to CCNU.
2. In all the tumours investigated, MISO enhanced CCNU cytotoxicity.
3. The resistant tumour showed enhancement only at high doses where the dose-limiting normal tissue was equally sensitized.
4. Sensitive tumours showed enhancement at lower CCNU doses where there was no sensitization by MISO of the cytotoxicity to marrow stem cells.
5. If sterility is considered to be an important factor in the treatment outcome, it should be noted that spermatogonia were sensitized by MISO almost as much as the tumours, at all CCNU doses.

This work was supported by research grants No. CA-15201 and CA-25990 from the National Cancer Institute, DHEW. The authors would also like to than the U.S. National Cancer Institute for supplying the misonidazole and SR-2508 used in these studies.

### REFERENCES

Barranco, S. C., Novak, J. K. & Humphrey, R. M. (1975) Studies on recovery from chemically induced damage in mammalian cells. *Cancer Res.*, **35**, 1194.

Brown, J. M., & Workman, P. (1980) Partition coefficient as a guide to the development of radiosensitizers which are less toxic than misonidazole. *Radiat. Res.*, **82**, 171.

Brown, J. M., Yu, N. Y., Brown, D. M. & Lee, W. L. (1981) SR-2508: A 2-nitro-imidazole amide which should be superior to misonidazole as a radiosensitizer for clinical use. *Int. J. Rad. Oncol. Biol. Phys.*, **7**, 695.

Clement, J. J., Gorman, M. S., Wodinsky, I., Catane, R. & Johnson, R. K. (1980) Enhancement of antitumour activity of alkylating agents by the radiation sensitizer misonidazole. *Cancer Res.*, **40**, 4165.

EWIG, R. A. G. & KOHN, K. W. (1978) DNA-protein cross-linking and DNA interstrand cross-linking by haloethylnitrosoureas in L1210 cells. *Cancer Res.*, **38**, 3197.

KALLMAN R. F., SILINI, G. & VAN PUTTEN, L. M. (1967) Factors influencing the quantitative estimation of the *in vivo* survival of cells from solid tumours. *J. Natl Cancer Inst.*, **39**, 539.

LAW, M. P., HIRST, D. G. & BROWN, J. M. (1981) The enhancing effect of misonidazole on the response of the RIF-1 tumour to cyclophosphamide. *Br. J. Cancer*, **44**, 208.

LU, C. C. & MEISTRICH, M. L. (1979) Cytotoxic effects of chemotherapeutic drugs on mouse testis cells. *Cancer Res.*, **39**, 3575.

MARTIN, W. M. C., MCNALLY, N. J. & DERONDE, J. (1981) The potentiation of cyclophosphamide cytotoxicity by misonidazole. *Br. J. Cancer*, **43**, 756.

MEISTRICH, M. L., HUNTER, N. R., SUZUKI, N., TROSTLE, P. K. & WITHERS, H. R. (1978) Gradual regeneration of mouse testicular stem cells after exposure to ionizing radiation. *Radiat. Res.*, **74**, 349.

MULCAHY, R. T., SIEMANN, D. W., & SUTHERLAND, R. M. (1981) *In vivo* response of KHT sarcomas to combined chemotherapy with misonidazole and BCNU. *Br. J. Cancer*, **43**, 93.

OAKBERG, E. F. (1956) Duration of spermatogenesis in the mouse and timing of stages of the cycle of the seminiferous epithelium. *Am. J. Anat.*, **99**, 507.

ROSE, C. M., MILLAR, J. L., PEACOCK, J. H., PHELPS, T. A. & STEPHENS, T. (1980) Differential enhancement of melphalan cytotoxicity in tumour and normal tissue by misonidazole. In *Radiation Sensitizers: Their Use in the Clinical Management of Cancer*, (Ed. Brady). New York: Manor Publishing. p. 405.

SIEMANN, D. W. (1981) *In vivo* combination of misonidazole and the chemotherapeutic agent CCNU. *Br. J. Cancer*, **43**, 367.

STEPHENS, T. C., COURTENAY, V. D., MILLS, J. PEACOCK, J. H., ROSE, C. M. & SPOONER, D. (1981) Enhanced cell killing in Lewis lung carcinoma and a human pancreatic-carcinoma xenograft by the combination of cytotoxic drugs and misonidazole. *Br. J. Cancer*, **43**, 451.

TANNOCK, I. F. (1980) *In vivo* interactions of anti-cancer drugs with Misonidazole or Metronidazole: Cyclophosphamide and BCNU. *Br. J. Cancer*, **42**, 871.

TILL, J. E. & MCCULLOCH, E. A. (1961) A direct measurement of the radiation sensitivity of normal mouse bone marrow cells. *Radiat. Res.*, **14**, 213.

TWENTYMAN, P. R. (1981) Modification of tumour and host response to cyclophosphamide by Misonidazole and WR-2721. *Br. J. Cancer*, **43**, 745.

WASSERMAN, T. H. (1974) Review of CCNU in clinical cancer therapy. *Cancer Treat Rev.*, **1**, 131.

WORKMAN, P. & BROWN, J. M. (1981) Structure–pharmacokinetic relationships for misonidazole and analogues in mice. *Cancer Chemother. Pharmacol.*, **6**, 39.